# Is There Neural Evidence for an Evidence Accumulation Process in Memory Decisions?

**DOI:** 10.3389/fnhum.2016.00093

**Published:** 2016-03-08

**Authors:** Marieke K. van Vugt, Marijke A. Beulen, Niels A. Taatgen

**Affiliations:** Cognitive Modeling Group, Institute of Artificial Intelligence and Cognitive Engineering, University of GroningenGroningen, Netherlands

**Keywords:** ECoG, iEEG, model-based neuroscience, brain oscillations, decision making, recognition memory

## Abstract

Models of evidence accumulation have been very successful at describing human decision making behavior. Recent years have also seen the first reports of neural correlates of this accumulation process. However, these studies have mostly focused on perceptual decision making tasks, ignoring the role of additional cognitive processes like memory retrieval that are crucial in real-world decisions. In this study, we tried to find a neural signature of evidence accumulation during a recognition memory task. To do this, we applied a method we have successfully used to localize evidence accumulation in scalp EEG during a perceptual decision making task. This time, however, we applied it to intracranial EEG recordings, which provide a much higher spatial resolution. We identified several brain areas where activity ramps up over time, but these neural patterns do not appear to be modulated by behavioral variables such as the amount of available evidence or response time. This casts doubt on the idea of evidence accumulation as a general decision-making mechanism underlying different types of decisions.

## 1. Introduction

Probably the most well-studied component of decisions is the process of evidence accumulation (Heekeren et al., 2008). Mathematical models that describe this evidence accumulation process, e.g., the drift diffusion model (Ratcliff, 1978), the linear ballistic accumulator (Brown and Heathcote, 2008), and leaky competing accumulators (Usher and McClelland, 2001) have been able to explain a wealth of behavioral data. Moreover, these models are increasingly used to elucidate the neural mechanisms underlying decision making. However, many of these studies have focused solely on simple perceptual decision making tasks, ignoring the fact that many real-life decisions and decision tasks also call for the retrieval of information from memory or other sources. Here we aim to extend this growing body of literature by looking at an accumulation process which uses information from memory in addition to perceptual sources. To do this, we use a method which has previously allowed us to find a signature of evidence accumulation in scalp EEG during a perceptual decision making task (random dot motion) (van Vugt et al., 2012). We apply this method to two-alternative forced-choice decisions in a recognition memory task.

Our approach is to select all brain areas where activity is roughly consistent with the time course of evidence accumulation, and then to apply further criteria to pinpoint each area's specific role in the process. Moreover, the results we present were obtained using intracranial EEG (iEEG), which has much better spatial resolution than conventional scalp EEG, combined with excellent temporal resolution (Jacobs and Kahana, 2010). However, iEEG has not previously been used to study the dynamics of evidence accumulation.

### 1.1. Modeling evidence accumulation

Accumulator models of decision making (Ditterich, 2010) assume that to make a decision, evidence is accumulated slowly over time, until the accumulated evidence reaches the threshold corresponding to a particular response, at which point the corresponding option is chosen. Several different implementations of such an evidence accumulation process have been conceived, but here we will focus on the Ratcliff Diffusion Model (DDM), which is widely used in the literature. While the recent neuroscience literature has focused on perceptual decision making, it is interesting to note that the DDM was originally used to model a recognition memory task (Ratcliff, 1978).

The DDM assumes that response time is determined by the time it takes the random walk process to reach a decision threshold, plus a fixed non-decision time reflecting perceptual processing of stimuli and preparation of a motor response. The speed with which the decision variable moves toward one of the boundaries, is influenced by the ambiguity of the evidence and quality of attention: stronger evidence and more focused attention will lead to a faster accumulation of evidence in favor of one particular option and thus to a faster decision. The height of the decision threshold reflects response caution or speed-accuracy trade-off. We will use all of these elements of the model as further criteria to narrow down the set of candidate neural accumulators.

### 1.2. Neural correlates of evidence accumulation

As reviewed by Heekeren et al. (2008), there is a consensus that for perceptual decision making, stimulus information is processed in sensory areas, then integrated or accumulated in parietal and frontal areas, and subsequently transmitted to effector areas to produce the desired responses. Indeed, single-unit studies in monkeys have found patterns of firing rate qualitatively consistent with evidence accumulation (Shadlen and Newsome, 2001; Gold and Shadlen, 2007; Churchland et al., 2011) in the lateral intraparietal area. Subsequent studies have shown similar neural patterns in the superior colliculus (Horwitz and Newsome, 2001), frontal eye field (Cohen et al., 2009; Purcell et al., 2010), caudate (Ding and Gold, 2010), and dorsolateral prefrontal cortex (Kim and Shadlen, 1999).

Neuroimaging studies in humans, which, unlike single-unit studies, are not limited to a single brain area, have replicated accumulation-like patterns for different types of sensory information in various frontal and parietal areas (e.g., Heekeren et al., 2006; Ploran et al., 2007; de Lange et al., 2010). While the effects observed in functional magnetic resonance imaging (MRI) were at best fairly indirect, due to the limited temporal resolution of this method, electrophysiological and magnetoencephalographic studies examining brain oscillations have also pointed at patterns that resemble those hypothesized by evidence accumulation models (e.g., Donner et al., 2009; van Vugt et al., 2012). Brain oscillations are widely believed to play an important role in our cognitive functioning (Buzsáki, 2006). During complex tasks that require the participation of several different, spatially separated brain areas, it is believed that synchronized oscillatory activity is what allows these neuronal populations to communicate and coordinate their functioning (Fries, 2005). In relation to evidence accumulation, studies making use of magnetoencephalography (MEG) have implicated 12–36 Hz beta and 64–100 Hz gamma in primarily effector regions (such as primary motor cortex) in this process (Donner et al., 2009). In contrast, EEG studies have implicated 4–9 Hz theta oscillations, which are also involved in more general decisional processes (Cavanagh et al., 2010; Womelsdorf et al., 2010), in evidence accumulation (van Vugt et al., 2012; Werkle-Bergner et al., 2014).

Importantly, all of the above-mentioned studies focus only on simple perceptual decisions, for which sensory input is often one-dimensional and administered to participants at a constant rate. The addition of a memory component in our task means that the neural implementation of evidence accumulation may be different than what was observed in perceptual studies. There have been a few studies that use sequentially presented cues containing information about an upcoming decision, where subjects need to remember a running average of the cues they have seen thus far. This has been done in both humans (Gluth et al., 2013) and monkeys (Kira et al., 2015). However, the role of memory in these tasks is very limited and both studies only look at one of the brain areas that may play a role in evidence accumulation. As a result, our knowledge of where this process takes place in a memory task is far from complete. It is possible that accumulation still takes place in the same brain areas as during a perceptual task, but with a slightly different time course, due to the additional time needed for memory retrieval. Alternatively, it is possible that additional or even different brain areas are now involved in the accumulation process. For this reason, we do not focus our analysis on any particular region of interest a priori, but look for correlates of evidence accumulation across the entire brain. Similarly, we do not focus on a single band of oscillatory activity but focus on all frequencies.

To preview our results, even with this data-driven approach, we were unable to find any signal that fits theoretical predictions for how an accumulator would behave. This raises the question whether evidence accumulation actually plays a role in more complex, memory-based decision tasks like the one used here, or if it does, how it is implemented in the brain. Our analysis assumes that the accumulation process is monotonic and linear, but maybe for this particular task, that is not a correct representation.

## 2. Materials and methods

### 2.1. Task

The task our participants performed used the Sternberg working memory paradigm (Sternberg, 1966) with faces and letters as stimuli. After fixation (1000–1075 ms, jittered), participants saw a sequence of 1–4 items for 700–775 ms each, separated by 275–350 ms interstimulus intervals. They remembered these items for a 3000–3300 ms retention period. After this interval, a probe item appeared and subjects were asked to judge whether this item was part of the memory set (a target) or not (a lure). Subsequently, the participants received accuracy feedback and pressed a key to advance to the next trial. The durations of stimulus presentations and intervals were jittered to avoid spurious correlations between ongoing brain activity and the timing of task events.

The memory sets participants studied consisted of either synthetic faces (see van Vugt et al., 2010) or consonants. Trials of each type were presented in blocks of 30 trials, with the order of blocks randomized across participants. For the current analysis, trials of both stimulus conditions (faces and letters) were combined into one large dataset. Only correctly answered trials with reaction times up to 2500ms were used for subsequent analysis.

In addition to varying study list length, the amount of decision evidence in face trials was further manipulated by systematically varying the similarity between faces in the memory set and the probe item. More precisely, the amount of decision evidence depends on summed similarity (Nosofsky, 1991). Summed similarity is the sum of the similarity values between the probe item and each of the items in the memory set. The higher the sum of these similarities, the more familiar a probe item feels, and consequently, the larger the probability it will be endorsed as a target. This means that for target trials, a high summed similarity corresponds to an easy trial with strong evidence for the correct decision. For lures, a higher summed similarity value corresponds to a more difficult trial.

### 2.2. Participants

The participants in this study were sixteen neurosurgical patients (ages 15–58; six female) who underwent surgery for pharmacologically intractable epilepsy. Patients had arrays of subdural and/or depth electrodes implanted to localize seizure onset and map cognitive functions prior to surgery. Electrode locations were based solely on medical considerations. Previous publications have reported on the neural correlates of memory processes in these data (van Vugt et al., 2009, 2010).

Patients were recruited from Brigham and Women's Hospital in Boston, the Hospital of the University of Pennsylvania in Philadelphia, and Universitäts Klinikum Freiburg in Germany, and the research protocol was approved by the appropriate institutional review boards at each hospital: the Partners Human Research Committee in Boston, the University of Pennsylvania Office of Regulatory Affairs Institutional Review Board and the Ethik-Komission der Albert-Ludwigs-Universität Freiburg, respectively. Written informed consent was obtained from all participants. In the case of minors, written consent was given by both the patient and their parents or custodians.

### 2.3. Intracranial EEG recordings

The intracranial EEG data were recorded by the clinical EEG system, and the task was administered by a separate laptop running the recognition memory experiment. We synchronized the testing and recording computers using optical pulses. By lining up these pulses we could determine the timing of events with a precision of < 4 ms.

The locations of implanted electrodes were determined from co-registered postoperative computed tomographies and preoperative MRI or from postoperative MRIs, and converted into MNI coordinates. Depth electrodes in the hippocampus were localized manually by clinicians by inspection of postoperative MRIs. Table S1 lists the number of electrodes located in each different Brodmann area, as well as the number of participants contributing these electrodes to each area.

### 2.4. Analysis of intracranial EEG data

The local field potential at each electrode was amplified and digitally recorded at sampling rates between 250 and 1024 Hz. Subsequently, line noise was removed using a Butterworth notch filter at 48–52 or 58–62 Hz, depending on the continent where data were recorded. Next, a series of artifact detection steps were performed on the data. This was done separately for each trial, such that all electrodes showing an artifact during a specific trial were excluded from later analysis for that trial.

The artifact detection process consisted of three steps. First, we normalized the amplitude of the local field potentials across all electrodes and trials, and for each trial, excluded all electrodes whose amplitude was more than four standard deviations removed from the mean at any point during the trial. Second, we excluded all electrodes in trials where their kurtosis exceeded a threshold of 4.5 (Delorme and Makeig, 2004). Finally, we excluded electrodes from trials where their variance exceeded 1.75 standard deviations above the population mean. These thresholds were determined empirically by visually identifying artifacts in data from a subset of participants and then finding the threshold values that optimize the number of correctly removed artifacts relative to the number of false alarms (defined as trials which the algorithm identified as artifacts while they were in fact usable). The resulting thresholds were then applied uniformly to the data of all participants.

For patients who participated in more than one recording session, the artifact detection process was performed separately for each session, to make sure our measures were not influenced by changes in the recording equipment between sessions.

After performing these three steps, all trials where more than 50% of electrodes showed any of the three possible artifacts, were removed from further analysis for all electrodes. Only participants who had at least 20 correctly answered letter- and 20 correctly answered face trials after artifact removal, were included in the rest of the analysis. This led to the removal of four participants, so all further analyses were performed on data from the 12 remaining participants. Similarly, electrodes that showed an artifact on more than 50% of trials in a particular recording session were removed from the dataset for that session. This led to the removal of an average of 8.08% of channels for the 12 participants included in our final analysis (SEM = 2.59%).

For each trial of the task, we calculated normalized oscillatory power using the Morlet wavelet transform with a width of 5. Normalization involved dividing each sample by the average and standard deviation across the 300-ms pre-trial baseline periods. We computed power at 53 frequencies, logarithmically spaced between 1 and 100 Hz. These were then divided into six different frequency bands: 2–4 Hz delta, 4–9 Hz theta, 9–14 Hz alpha, 14–28 Hz beta, 28–48 Hz low gamma, and 48–90 Hz high gamma (van Vugt et al., 2010). Datasets with very high sampling frequencies were downsampled to 250 Hz prior to wavelet transform. Afterwards, all wavelet-transformed time series were downsampled again to 50 Hz to give all participants' datasets the same sampling rate and to reduce the computational burden. The same was done with the raw EEG signal (van Vugt et al., 2012).

### 2.5. Patterns of brain activity

Theories about evidence accumulation predict a specific pattern of activity to be present in the brain during decision making. We translated this predicted pattern of activity into a regressor and tested how well that could predict the brain activity we recorded (i.e., the amplitude of the raw EEG and the amplitude of wavelet-transformed EEG in different frequency bands). We followed the same procedure that was used in our previous study of scalp EEG activity during a perceptual decision making task (van Vugt et al., 2012). In the first step of our analysis, we used linear regression to find the extent to which the EEG signal at each electrode matched the predicted patterns.

**Figure 1:**
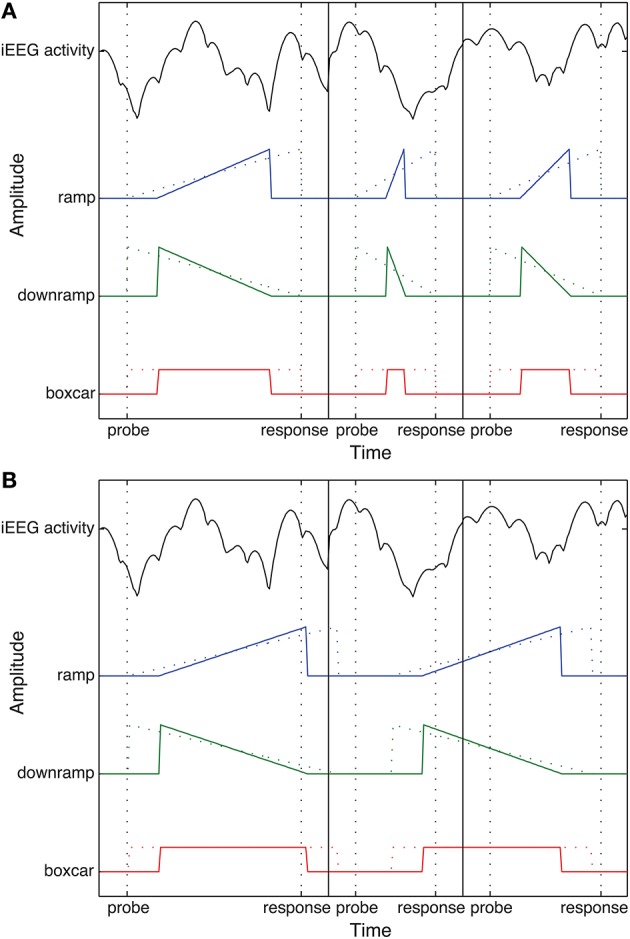
**(A)** Example time courses of one electrode's theta activity and the matching ramp, downramp, and boxcar regressors for three consecutive trials. **(B)** Example time courses of “random” ramp, downramp and boxcar regressors: made using randomly shuffled reaction times. Note that in this case, onset and offset of the ramp no longer correspond to “probe” and “response,” respectively. Dotted colored lines show time course of regressors before correction for non-decision time. Vertical lines indicate borders between real task trials.

Specifically, a neural correlate of evidence accumulation would be a type of activity that starts at zero when the probe stimulus is shown and decision evidence first becomes available, gradually keeps building up until a decision is made and then drops down to a baseline level again. We simulated this as “ramps” that build up from 0 to 1 over the duration of each decision period (the interval from the onset of the probe stimulus until the response), and are 0 at all other times. The lengths of these ramps match the durations of each trial that subjects actually performed (i.e., their individual reaction times). An example is shown in Figure 1.

The DDM predicts that a portion of the time between the onset of the probe and the response is taken up by the non-decision time, which reflects perceptual and motor delays. To account for this, the durations of each participant's ramps were adjusted for their individual non-decision time as estimated by the Robust EZ package for R (Wagenmakers et al., 2008). This was done so that half of the non-decision time comes before the onset of the ramp, reflecting non-decision processes that take place before the start of evidence accumulation (e.g., stimulus processing), and the other half after the ramp has returned to baseline, reflecting non-decision processes between accumulation and response execution.

To see where in the brain our ramping pattern is the best predictor of activity during decision periods, we contrasted the ramps to two alternative patterns of activity that are not consistent with evidence accumulation (see also Figure 1). In the first alternative, activity increases sharply at the beginning of the decision period and them slowly declines back to baseline, forming a downward ramp^1^. The second alternative is a “boxcar” pattern: a constant level of increased activity during the entire decision period, corresponding, for example, to a generic “attention on” state, which also returns to baseline when the decision is made (e.g., Raghavachari et al., 2001). To model this departure from and return to baseline as well, all regressors also contain 300 ms of a signal with zero amplitude before and after the decision interval.

The dependent variable in our regression analysis consists of the ampitude of oscillatory power in each of the seven frequency bands, recorded during the decision period of each trial, with a 300 ms buffer of extra-trial time on each side. For each electrode, the decision intervals from all the trials the participant performed were combined into one long dependent variable that exactly matches the length of the regressors for that participant (see Figure 1A, top, for illustration). Trials containing artifacts were excluded from these features, as well as the corresponding regressors.

To compare the oscillatory frequency bands, we made seven separate features for each electrode, containing the time courses of oscillatory activity in each of the frequency bands and of the raw EEG amplitude. Each of these features was analyzed separately in regression analyses with each of the three regressors.

**Figure 2:**
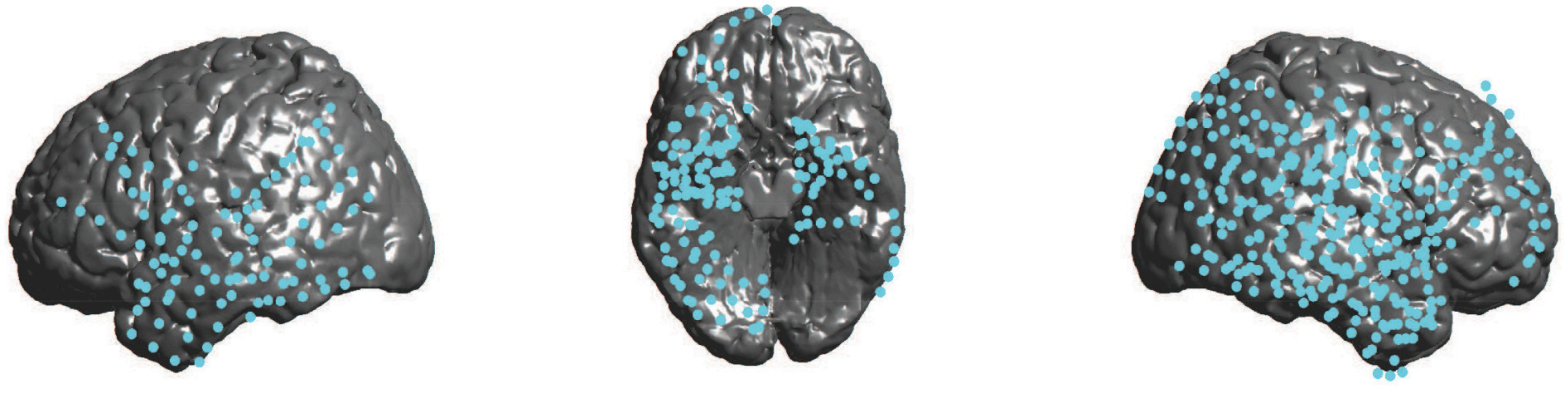
**Locations of all cortical electrodes in our dataset**.

For the next step in our analysis, we grouped all electrodes according to the Brodmann area in which they were located, to determine which brain areas in particular show activity representative of evidence accumulation. In total, we recorded electrodes from 34 different brain areas. For our statistical analysis, we only included brain areas for which we had at least 10 electrodes, to avoid spurious conclusions based on too little data. This led to the inclusion of 25 Brodmann areas (see Table S1). Figure 2 shows the locations of all 852 electrodes included in our final analysis.

### 2.6. Assessing significance with random regressors

As a first investigation of whether the ramping pattern our model predicts is visible in the brain when a decision is made, we used a canonical correlation analysis (CCA, van Vugt et al., 2012). The benefit of this method is that it tells us to what extent ramp-like activity is present in the activity of all electrodes combined, instead of limiting the search to a single electrode at a time (similar reasoning is used in MVPA for fMRI data, see e.g., Norman et al., 2006). This leaves room for the possibility that accumulation takes place in a combination of different brain areas at different times during the decision process. The CCA analysis gives a single value for the “fit” of each regressor to certain frequencies of activity across the entire brain. This allows us to see whether there is any neural pattern that shows the predicted ramping activity.

Specifically, the CCA analysis looks for correlations between two sets of variables by identifying linear combinations of the variables in one set that optimally predict (combinations of) variables in the other set. In our case, a set of independent variables is formed by all the time courses of a certain type of activity, e.g., the features containing the theta activity of all electrodes. The analysis makes a weighted linear combination of all electrodes' time courses, so that if a brain area is not involved in evidence accumulation at all and consequently does not show ramp-like activity, it is possible that electrodes overlying that area receive a low weight and contribute little to the final correlation. In other words, CCA does not assume or require that the entire brain is involved, but it finds the relevant brain areas in a data-driven way.

As in our previous study, we performed a separate CCA for each regressor (upramp, downramp, and boxcar). The main outcome of the CCA, in this case, is the strength of the resulting overall correlation. Since it was not possible to combine electrodes across participants, as their locations are not standardized like in scalp EEG, we performed the CCA for each participant separately and then Fisher-transformed and averaged the resulting canonical correlations within each Brodmann area such that every participant contributed equally.

To assess significance of these canonical correlations, and to ensure that the ramping we observed was not a statistical artifact, we performed a permutation analysis in which the durations of the trials in our regressors were randomly rearranged so they no longer started and ended with the decision periods in the EEG data (see Figure 1B). Then, the canonical correlation with the EEG data was obtained for these rearranged regressors. This procedure was repeated 1000 times for each regressor type, frequency band and participant, and the resulting correlations were then averaged across participants. The correlation between the “real” ramp regressor and our EEG data was then compared to this distribution of correlations obtained without temporal alignment. Significant correlations should exceed the correlations derived from random data.

### 2.7. Linear mixed effects model to find accumulator location

After testing whether there was any ramp-like activity in the brain in general, we wanted to see in more detail where this activity was taking place. For this, we used a linear mixed effects model (LME-model; Pinheiro and Bates, 2009). This modeling technique takes into account the hierarchical nature of the data (where electrodes are nested within participants), and can deal with imbalances in the data (e.g., not all Brodmann areas contain electrodes from all participants). The mixed effects models included the fixed effects of frequency band, regressor type and brain area, as well as random effects of subject and individual electrode. This allowed us to examine what areas and frequencies show a larger ramping effect than average, without having to perform many individual comparisons. The LME-model was implemented using the lmer function of the lme4 package (Baayen et al., 2008) in the statistical software R.

The dependent variable in our LME model were simple linear regression of each electrode's seven features against each of the three regressors. We then asked what Brodmann areas and frequency bands the ramp regressor is a significantly better (i.e., higher regression slope) than average fit to the EEG data.

Since we were only interested in whether a specific frequency band in a brain area was correlated with the ramp regressor more than average, we only included interactions of brain area and frequency band in the model. This means that the LME-model contains a single fixed-effect independent variable: the interaction term of brain area and frequency band (see the equation below). In addition, since there was no clear reference level for either factor, we removed the intercept. Finally, model comparisons showed that the best fitting model included random intercepts for individual participants and electrodes. This led to a model described by the following equation:

(1) $$\begin{array}{r} corr=-1+\beta_{1_{i,j}}band_{i}\times area_{j}+\;\beta_{2_{subj}\text{r}}+\beta_{3_{elec}\text{r}} \end{array}$$

where *corr* = Fisher-transformed, centered correlation coefficient for each electrode at each frequency band; *band* = frequency band; *area* = Brodmann area; *subj* = participant number; *elec* = unique (across participants) electrode number; β = fitted model coefficients.

Before being entered into the analysis, the correlation coefficients were first Fisher-transformed, and then centered by subtracting the mean of all correlation coefficients. As a result, the model coefficient for each band-area pair refers to the deviation of that pair's correlation with the ramp regressor from the mean of all tested correlations. We then tested which of these coefficients significantly differed from zero using the *t*-test implemented in the cftest function of the multcomp (Hothorn et al., 2008) package in R. This function tests whether model coefficients are different from zero, while controlling for multiple comparisons.

After identifying the brain area and frequency band pairs that best match the ramp regressor, we ran separate LME-models within each of them to compare the fit of the ramp regressor to that of the downramp and boxcar regressors. Each of these models is described by the following equation:

(2) $$\begin{array}{r} corr=\beta_{0}+\beta_{1_{i}}regr_{i}+\;\beta_{2_{subj}\text{r}}+\beta_{3_{elec}\text{r}} \end{array}$$

where *corr* = Fisher-transformed, centered correlation coefficient for each electrode and regressor at each frequency band; *regr* = regressor type; *subj* = participant number; *elec* = unique (across participants) electrode number; β = fitted model coefficients.

The resulting *p*-values were corrected for multiple comparisons using a false discovery rate (FDR) procedure. In contrast to methods like Bonferroni correction, which are very conservative for large numbers of comparisons, the FDR procedure works by controlling the total proportion of false positives in a complete set of comparisons.

After this correction, we selected the area and band combinations for which the ramp regressor provided at least an equally good fit as the other two patterns (boxcar and downramp).

### 2.8. Event-related averages to test for more specific accumulator properties

All combinations of brain areas and frequency bands where the ramp regressor showed an above-average fit in the LME-model analysis, were marked as candidates for evidence accumulation. We then tested if each of these areas and bands fulfilled further theoretical predictions for accumulators. This was done by inspecting the time courses of their activity during the decision period.

Since there were large differences in reaction times between trials, even after grouping them into bins, event-related averages within each reaction time bin were vincentized to obtain a more reliable time course (Workman and Adams, 1950). In this method, each trial is resampled to the same number of samples, in this case the median reaction time in each bin. Short trials are stretched, while long trials are compacted. The benefit of this method is that it ensures that processes happening during a specific portion of each trial are not obscured by averaging over trials of very different lengths. Processes that take a fixed amount of time on each trial, like initial stimulus processing or response preparation, may become over- or underrepresented in the vincentized version of a trial. However, given that we have relatively long reaction times of which these non-decision processes form a relatively short fraction, and we are most interested in what happens in between, we believe this is not a problem.

## 3. Results

### 3.1. Behavioral results

**Figure 3:**
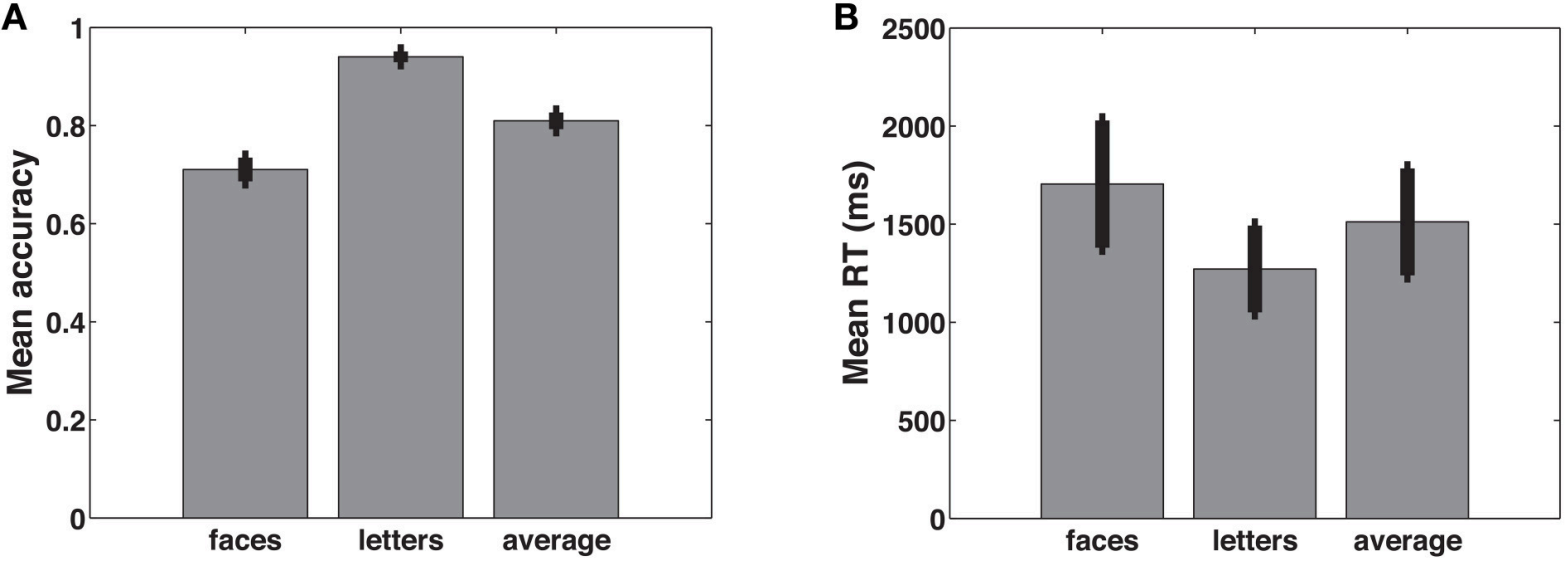
**Mean accuracy (A) and reaction times (B) for faces, letters, and the average of the two**. Error bars represent the standard error of the mean.

We first examined behavioral performance in the delayed recognition task (Figure 3). Mean accuracy was 81.0% (SEM = 1.7%) for letters and faces combined; mean reaction time was 1512 ms (SEM = 273 ms). Performance was slightly better on trials with letters than with faces [lower RT: *t*_(11)_ = −3.1049, *p* = 0.01; higher accuracy: *t*_(11)_ = 11.9158, *p* < 0.001]. Nevertheless, in subsequent analyses, we combined face and letter trials because according to the theory, a similar evidence accumulation process should occur for both types of stimuli.

**Figure 4:**
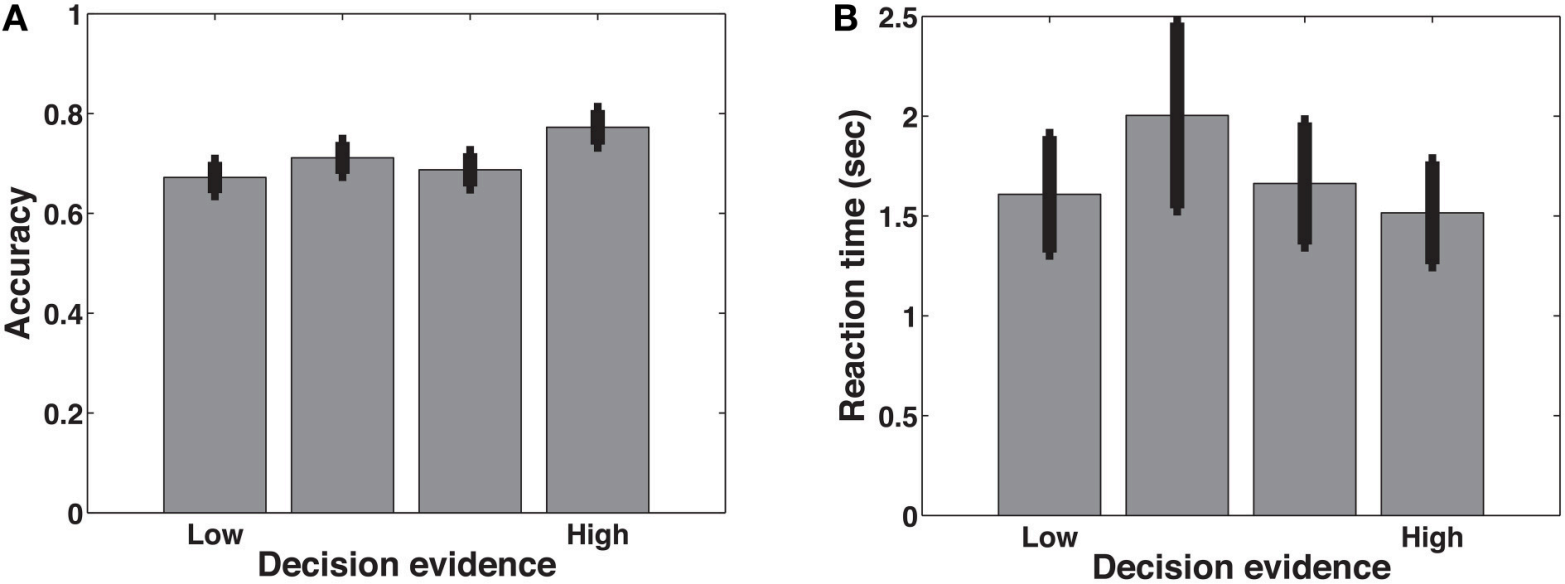
**Mean accuracy (A) and reaction times (B) separated by decision evidence as indexed by summed similarity**. Error bars represent the standard error of the mean.

The only case where we did not combine face and letter trials is the comparison between trials of low and high summed similarity. Summed similarity is the sum of the amount of similarity between the probe stimulus and each of the list stimuli. Previous work has shown that this is a measure of task difficulty (Nosofsky, 1991; Kahana and Sekuler, 2002). Note that this manipulation only applies to face trials, for which similarity distances were systematically manipulated. As expected (Figure 4), higher levels of decision evidence were associated with higher accuracy and faster response times, except for the lowest (most difficult) level, where participants may have resorted to guessing.

As described in Materials and Methods above, we estimated each participant's non-decision time using the Robust EZ package (Wagenmakers et al., 2008). The mean estimated non-decision time was 0.63 s, with a standard deviation of 0.17 s.

### 3.2. Ramp-like activity in the brain

**Figure 5:**
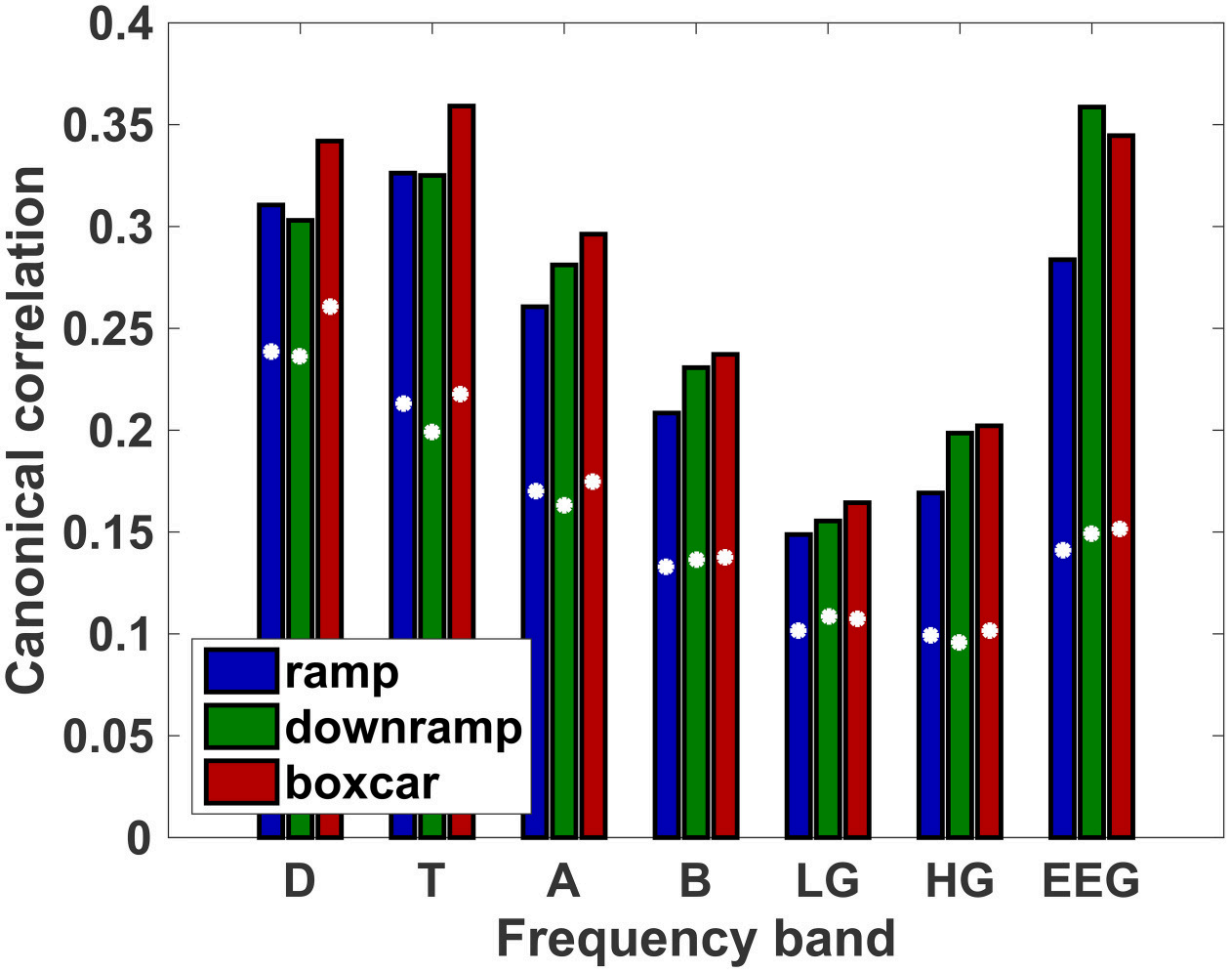
**Mean canonical correlation between each regressor and the time course of whole-brain EEG/oscillatory power**. White dots indicate 99th percentile of correlations obtained from regressors with randomized timing (i.e., they represent the chance level of each correlation).

To see if there was any ramp-like activity in the brain that was significantly time-locked to our task, we performed a canonical correlation of each regressor against the activity of all electrodes. Figure 5 shows that the correlation of our task-timed ramp regressor is above 99% of the distribution of correlations obtained with randomized timing in all frequency bands. The highest correlations are observed in the delta and theta bands, as well as for the raw EEG signal. The same, however, is true for the downramp and boxcar regressors. In other words, our ramp regressor does not seem to describe overall brain activity during decision periods better than the two alternative patterns. However, this does not mean that all three regressors are associated with the same neural patterns. It is very well possible that ramp-like and boxcar-like activity take place simultaneously in different parts of the brain. To disentangle timing from localization of ramp-like and boxcar-like activity, we performed regressions in individual Brodmann areas.

### 3.3. Brodmann areas that show a ramping pattern

To examine where in the brain the ramp-like and boxcar-like activity demonstrated by the previous analysis take place, we used a linear mixed effects (LME) model to find which areas and frequency bands activity was more ramp-like than boxcar-like.

**Figure 6:**
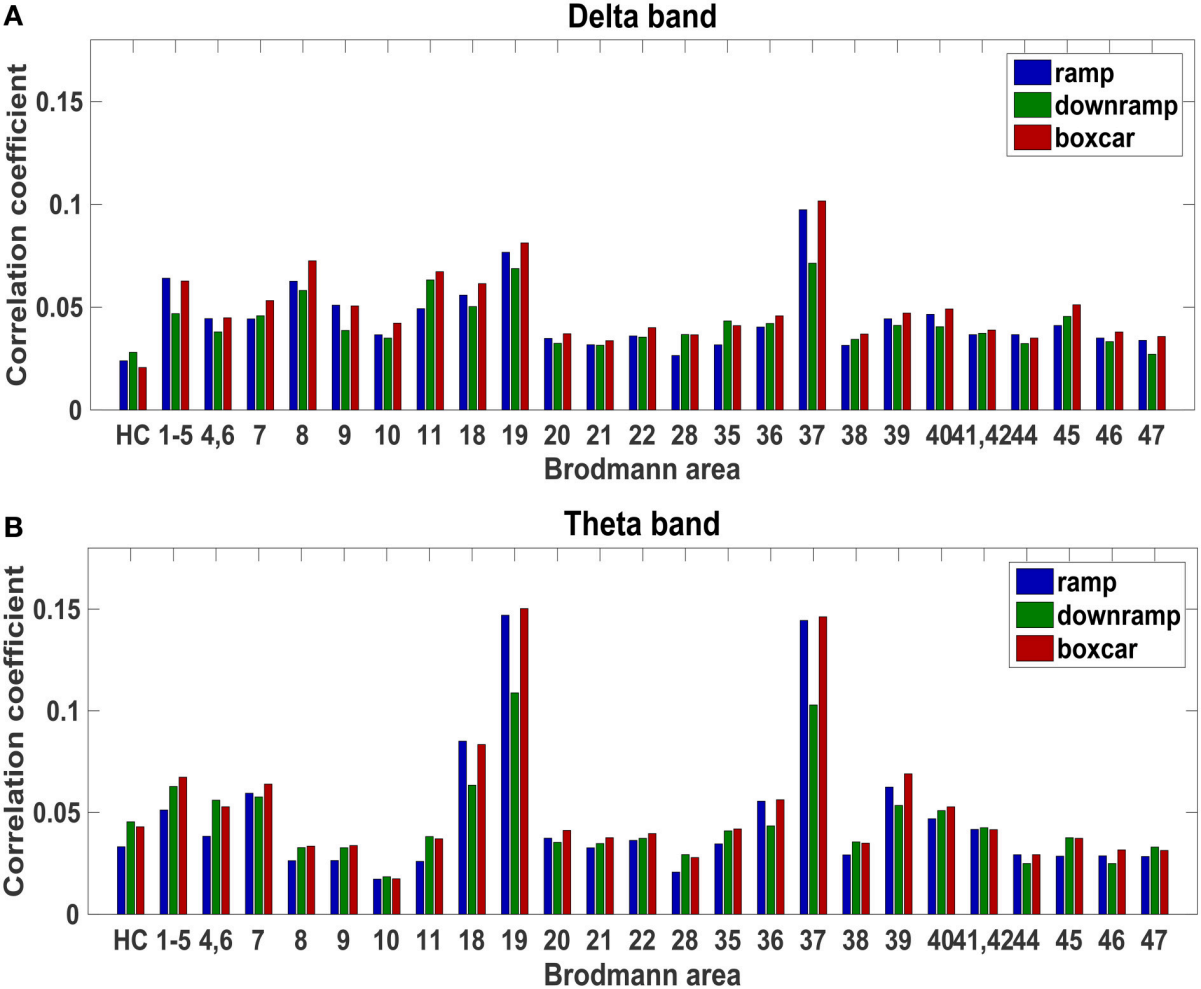
**Correlation of regressors with 2–4 Hz delta (A) and 4–9 Hz theta (B) activity for each Brodmann area**. HC, hippocampus; 1–5, Brodmann areas 1, 2, 3 & 5; 4,6, Brodmann areas 4 & 6. Ideally, neural accumulator candidates should have a higher correlation with the ramp regressor (blue) than with the downramp (green) and boxcar (red) regressors.

As shown in Table 1, four regions show significant ramping in low frequency bands: Brodmann areas 1, 2, 3, & 5 (somatosensory cortex), 9 (dorsolateral prefrontal cortex), and 37 (occipitotemporal cortex; inferior temporal gyrus and fusiform gyrus) in the 2–4 Hz delta band, and Brodmann areas 19 (occipital cortex; visual association cortex) and 37 in the 4–9 Hz theta band. The correlation (averaged over patients) of the three regressors with delta and theta activity in each brain area is shown in Figure 6. We are looking for areas that show the ramping pattern (blue line) more than other areas, but also at least as clearly as the two alternative patterns. The figure demonstrates that our five areas satisfy this criterion.

**Table 1:** **Brodmann areas and frequency bands which show significantly higher correlations with the ramp regressor than with the other regressors**.

**Brain area**	**Frequency band**	**Estimate**	***p*-value**
BA1,2,3,5	delta (2–4 Hz)	0.033	1.21e-05
BA9	delta (2–4 Hz)	0.025	7.13e-05
BA19	theta (4–9 Hz)	0.110	<2e-16
BA37	delta (2–4 Hz)	0.063	<2e-16
BA37	theta (4–9 Hz)	0.109	<2e-16

Estimate shows the deviation of the correlation with the ramp regressor from the grand mean. BA, Brodmann area; BA1,2,3,5, somatosensory cortex; BA9, prefrontal cortex; BA19, occipital cortex; BA37, occipitotemporal cortex. P-values were obtained from multiple-comparisons-corrected t-tests.

In addition to these five area–band pairs, there were 11 pairs where the correlation with the downramp regressor did not significantly differ from that with the ramp regressor. Because we were specifically interested in areas that showed ramp-like activity and *did not* show a downramp-like pattern of activity, we did not include these areas in our analysis. We report these pairs in Table S2. There were no brain areas where the ramp regressor fitted the neural data significantly better than the boxcar regressor. Even though this is some reason for worry about the areas being true accumulators, we still proceed to inspect the time courses to check whether our candidates are true accumulators. To preview our results, these suggested that in fact there was little support for that idea.

### 3.4. Time courses of activity

**Figure 7:**
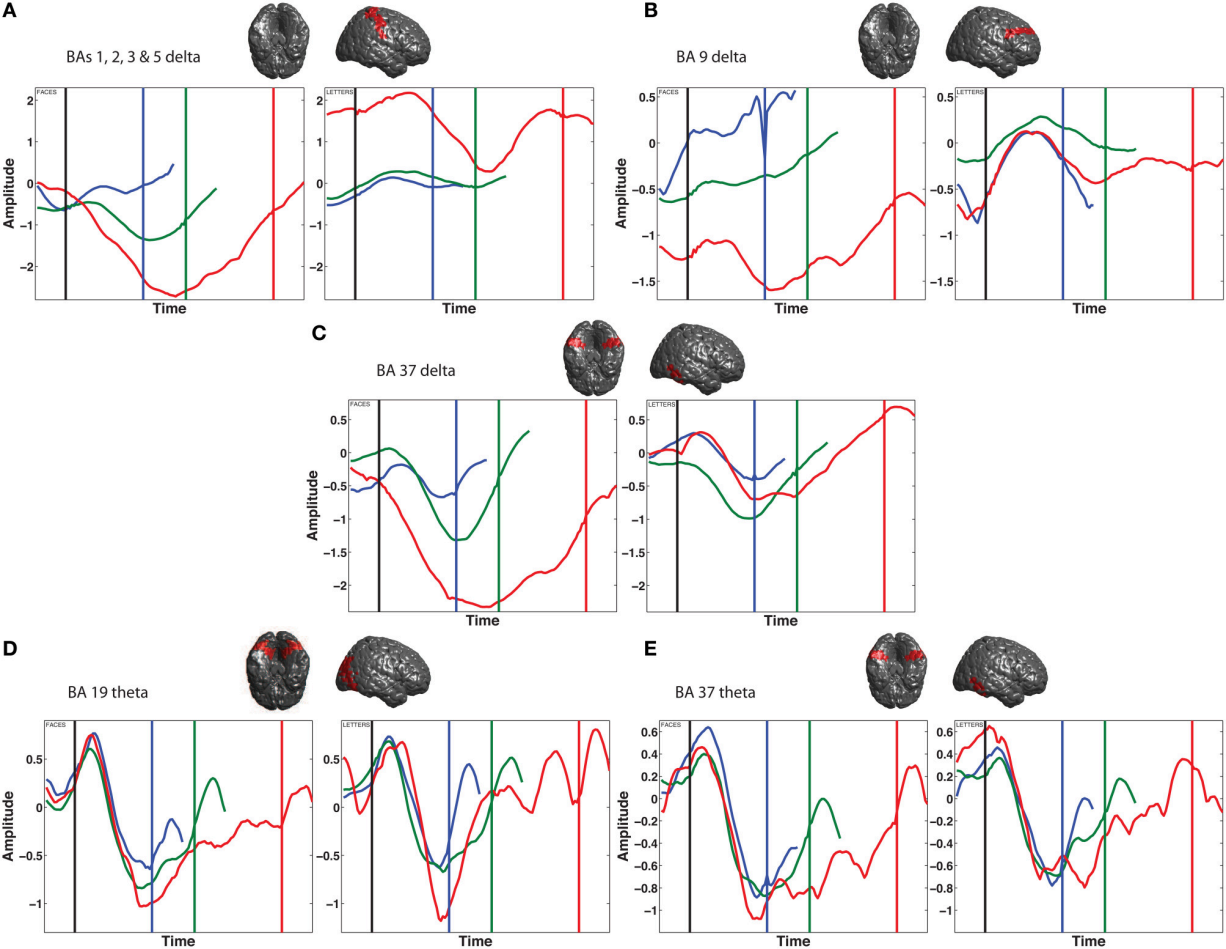
**Time course of normalized power for short (blue; RT < 900 ms), medium (green; 900 < RT < 1700 ms) and long (red; RT > 1700 ms) reaction time (RT) bins**. Left panels show face trials, right panels show letters. Top rows: 2–4 Hz delta power; **(A)** Brodmann areas 1, 2, 3 & 5; **(B)** Brodmann area 9; **(C)** Brodmann area 37. Bottom row: 4–9 Hz theta power; **(D)** Brodmann area 19; **(E)** Brodmann area 37. Vertical lines represent probe onset (black) and response (blue, green, and red). Plots are vincentized: individual trials have been resampled to a single duration to facilitate averaging over trials of different lengths.

Figure 7 shows average vincentized time courses of delta and theta power in the areas that showed significant ramp-like activity in these bands (according to Table 1). Since some of these areas respond very differently to trials with faces or letters as stimuli, these two categories are shown separately.

Some areas show a small positive peak at stimulus onset, but after that, power gradually moves away from the baseline level (zero) in all areas. The power then returns to baseline around the response. This is consistent with the behavior expected from a neural evidence accumulator. However, the ramping activity in all areas and frequency bands except prefrontal (BA9) delta goes downward rather than upward from baseline, signaling a decrease in power over the course of the trial.

A further criterion for a neural accumulator is that the peak of the accumulation should be reached later on trials with longer reaction times (O'Connell et al., 2012). To examine this, we compared the activity in our areas of interest between three different reaction time bins, where short trials were defined as reaction times under 900 ms, and long trials as reaction times over 1700 ms (Figure 7). In the theta band, in both areas it is clear that the ramps peak at exactly the same moment regardless of response time. In the delta band, the exact timing of the return to baseline is somewhat different for different reaction time bins, but none of them show a ramp that continues all the way up to the response for all three bins.

If this ramping signal triggers a response, the amplitude at which it peaks should correspond to the decision threshold so this peak magnitude should be independent of response time (O'Connell et al., 2012). Our neural ramps do not seem to stop at the same level in all cases. Instead, in the areas where ramp duration scales somewhat with response time (delta power in BAs 1, 2, 3 & 5, and 37), the peak amplitude for longer trials is much higher than for short trials. To a lesser extent, the same holds true for theta power in BAs 19 and 37.

A surprising finding was that Brodmann areas 1, 2, 3 & 5 and Brodmann area 9 showed qualitatively different responses to face and letter trials, only showing an accumulation-like process in one of them (in face trials for BAs 1, 2, 3, & 5 for face trials, and in BA 9 for letter trials), whereas the response to the other stimulus type looks fairly random. Based on theories of evidence accumulation, we were looking for a domain-general accumulator (Ho et al., 2009), which is independent of the type of stimuli being processed. Indeed, the other three band–area pairs we found all showed very similar responses to face and letter trials. While BAs 1, 2, 3 & 5, and 9 were also identified as accumulators in the combined analysis, they showed very different responses to the two types of stimuli, only showing the characteristic gradual move away from baseline for one stimulus type. A direct comparison of these responses for all reaction times combined can be seen in Figure S1.

Our next criterion was that the ramping activity in our candidate accumulators should be modulated by decision evidence. The DDM predicts that on more difficult trials, where the evidence is less clear, the rate of evidence accumulation will be lower. Conversely, if our neural ramps represent evidence accumulation, they should be steeper during easier trials (e.g., Donner et al., 2009). We manipulated decision evidence with summed similarity (see Materials and Methods) and list length.

**Figure 8:**
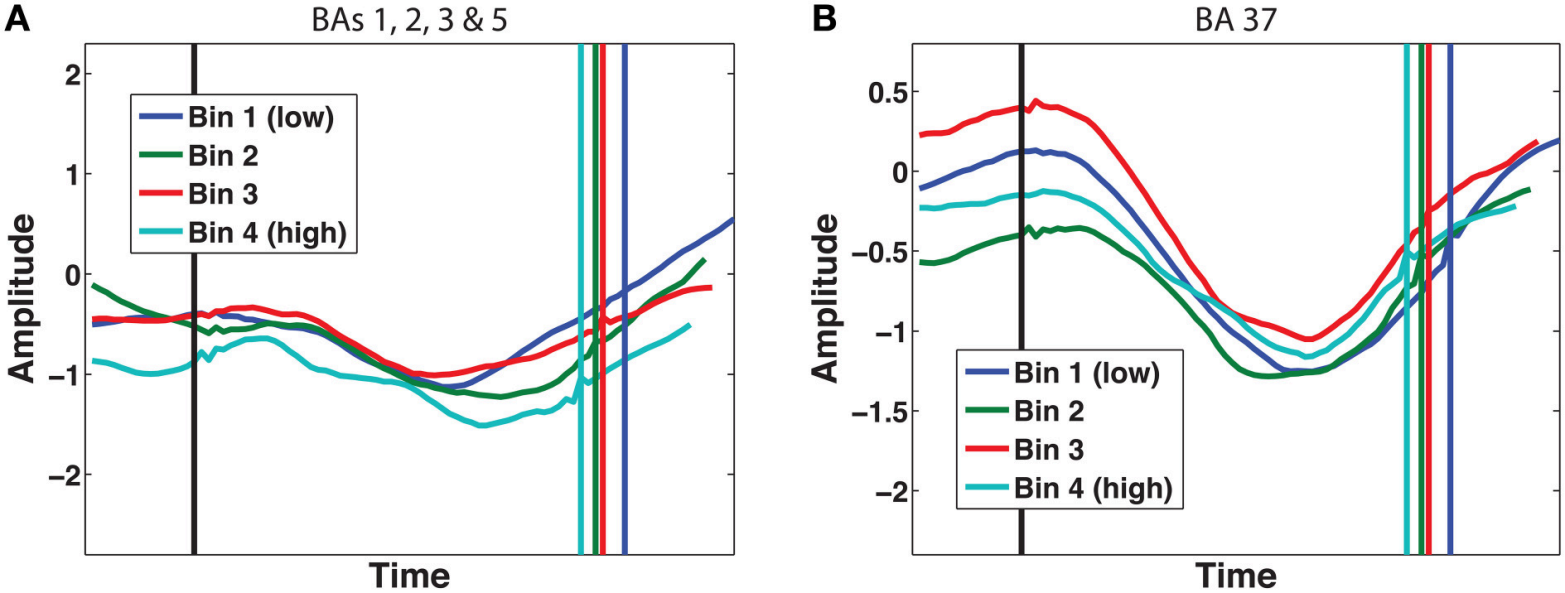
**Time course of normalized power for different levels of decision evidence (summed similarity)**. **(A)** 2–4 Hz delta power in Brodmann areas 1, 2, 3 & 5; **(B)** 2–4 Hz delta power in Brodmann area 37. Vertical lines represent probe onset and response. Plots are vincentized such that individual trials are resampled to a single duration to facilitate averaging over trials of different lengths.

The manipulation of decision evidence with summed similarity was only possible for face trials, and since Brodmann area 9 did not show clear ramping during face trials, we only show results for the other two areas where we found ramp-like delta-band activity: BAs 1, 2, 3 & 5, and 37. As can be seen in Figure 8, neither of these two areas show a consistent scaling of activity with the amount of available decision evidence.

Similarly, we also compared trials of different list lengths for both face and letter trials, as another manipulation of evidence strength. Again, none of the signals identified by our LME-model show a systematic decrease in slope from easy to difficult trials.

## 4. Discussion

In this study, we set out to find whether neural correlates of evidence accumulation can be observed in the brain during two-alternative forced choice decisions about recognition of remembered stimuli, and if they are similar to what has previously been found in perceptual decision making studies. Although we tried very hard, using previously-validated methods, to find such neural accumulators, we could not find them. We observed that the amplitude of low-frequency oscillations ramped up from probe onset until the response in four different brain areas (Brodmann areas 1, 2, 3 & 5, 9, 19, and 37). However, none of these signals fit our more specific criteria for accumulators such as scaling with response time or trial difficulty.

### 4.1. Potential confounds due to epileptic activity

There are several potential reasons for not finding a neural correlate of evidence accumulation in our memory task. First, it may be an artifact of the epilepsy in our participant sample. Second, we may not have the correct brain coverage. Third, the non-decision time we use to offset our hypothesized signal may be incorrect. We will discuss each of these possibilities in turn.

In this study, our participants were not the healthy 18–25 year old college students who often participate in research studies (Henrich et al., 2010). Instead, our participants were patients of different ages and educational backgrounds, who suffer from severe epilepsy. It may seem reasonable to attribute the absence of results to their pathology. However, we have taken extensive measures to remove epileptic artifacts from our data, and the participants included in our study all performed the task well. In addition, our analysis only included Brodmann areas that we measured in multiple participants. Hence we believe that the signals we report here are representative of activity in healthy brains.

The fact that patients participated voluntarily between medical treatments meant that some patients were not able to perform as many trials as others. When time courses of activity are separated into several difficulty or reaction time bins, this means that some patients contribute only a few trials per bin. As a result, these time courses are quite noisy, and it is possible that this noise obscures existing effects of reaction time or difficulty. However, if this were the case, one would expect to see at least a trend, and even this is not present in most of the areas we looked at. As a result, we doubt if additional trials would have led to any more clearcut effects in these cases.

### 4.2. Coverage of brain areas

Since the placement of electrodes was entirely based on medical considerations, we were not able to ensure equal coverage of all brain areas. As can be seen in Figure 2, especially frontal and parietal cortex were not completely covered. This includes several areas where one might expect evidence accumulation to be found. Examples are parts of parietal cortex such as intraparietal sulcus, previously found to respond to strength of evidence in a perceptual decision task (Heekeren et al., 2008), anterior cingulate cortex, which is well-known to be involved in decision making (e.g., Cohen et al., 2007), or posterior cingulate cortex, which was also identified by Heekeren et al. (2006) as a possible neural accumulator. We cannot rule out that these areas would have shown the signal that we were looking for.

Nevertheless, we did have data from several other areas that have been named as sites of evidence accumulation in previous studies, and would therefore also be expected to exhibit neural correlates of accumulation. As described above, single-unit studies in monkeys have identified the lateral intraparietal area and dorsolateral prefrontal cortex (corresponding to BA's 7 and 9), among others, as neural accumulators. In humans, Ploran et al. (2007) used fMRI to investigate which role different brain areas play in different sub-processes of perceptual decision making. They identified a series of accumulator areas that integrate evidence over time, including Brodmann areas 7, 9, 19, and 37 and several other areas we also measured. In addition, in our previous perceptual decision making study, we also found a signature of evidence accumulation in parietal and occipital electrodes in scalp EEG (van Vugt et al., 2012), likely coming from areas that we also measured in the current experiment. It is striking that even in these areas, we did not find a clear signature of evidence accumulation anywhere in our current experiment. Moreover, preliminary findings from a follow-up study using scalp EEG also did not show any neural signature of evidence accumulation using the same methodology in a similar face recognition task. Taken together, this makes it more likely that the specific linear ramping pattern we were looking for simply is not present in the brain during such a task.

### 4.3. Potential artifacts of correcting for non-decision time

In the analysis described above, the duration of the ramp regressor on each trial was equal to the duration of that trial minus that participant's estimated non-decision time, with non-decision time being distributed equally before and after the ramp. We chose this approach because both before and after the accumulation process, the decision window likely also includes some other processes, such as stimulus processing and response preparation. However, to examine to what extent shortening the ramps influenced our results, we performed the same analysis with ramps that spanned the entire period from probe onset to response. In this case, the LME model again identified significant ramping in theta oscillations in Brodmann areas 19 and 37, but also in theta oscillations in BA 7 and, instead of low-frequency oscillations, in the high gamma (48–90 Hz) band in BA 9. We also looked at event-related activity in these two additional band-area pairs, but again, there was no evidence of scaling with response time or trial difficulty. Therefore, we chose not to show these results in more detail here.

### 4.4. What does the ramping activity reflect?

Although the ramping activity we observed did not show all the predicted characteristics of evidence accumulation, it is significantly time-locked to the decision period of our task. This leaves the question of what role this activity plays in the decision process. The theta activity in occipito-temporal areas 19 and 37, we believe, is most probably related to processing the stimulus on the screen. Such a sensory process is likely to be triggered by stimulus onset and independent of response time (Ploran et al., 2007), just like the theta activity we observe in these areas (Figure 7, bottom row). As for somatosensory and prefrontal cortex (Figure 7, top row), the ramping up of low-frequency oscillations there may reflect “tuning in” to the sensory evidence coming from posterior areas (perhaps communicated via delta oscillations in BA37, which show a time course very similar to BAs 1, 2, 3, and 5). The subsequent return to baseline, sometimes long before the response is executed, may reflect a shift away from this communication with visual areas and back to a more internal focus, reflecting the fact that the influence of new incoming visual evidence on a decision becomes smaller and smaller as more evidence has already been collected.

### 4.5. Implications

Of course, this leaves open the question where the final step of evidence accumulation which actually triggers the response, takes place. There are several possible explanations for our failure to find a neural signature of this process in this study.

First of all, it is possible that a linear, monotonic evidence accumulation process like the one represented by our ramps takes place in this task, but our recordings failed to capture it. As described above, using intracranial EEG has had drawbacks with regard to the coverage of different brain areas and the number of trials that could be collected, but we believe that if such a process took place, we would nevertheless have observed (part of) it.

Another possibility is that there simply is no evidence accumulation during a memory task, and memory-based decisions are made by a completely different mechanism than perceptual decisions. It would be far too soon to draw such a conclusion at this point. Moreover, it does not seem likely that the brain would have evolved completely separate mechanisms for these two very similar types of decisions.

Instead, the most likely conclusion is that there is evidence accumulation in the brain during this task, but it does not match the assumptions we made in our analysis. For example, the ramping up of activity from probe onset to decision may not be linear when evidence is not presented in a gradual way, or activity may not return to baseline right after the response as we predicted. If this were the case, our current analysis method would not pick up such a process. To investigate this possibility, future studies should make fewer assumptions about the time course of evidence accumulation in a memory task. Instead of looking for a specific time course of activity, it may be better to first look for brain areas that are sensitive to the amount of evidence for a decision and then let the data show how this activity develops over time.

### 4.6. Data sharing

The raw iEEG data are available on http://memory.psych.upenn.edu/Request_EEG_access?paper=vanVEtal12.

## Author contributions

Mv conceived of the study, oversaw the analysis and wrote the paper. MB performed the analysis and wrote the paper. NT oversaw the analysis and wrote the paper. All approved of the final version of the paper.

## Funding

Marie Curie Career Integration Grant ACCDECMEM to Mv, ERC starting grant MULTITASK to NT (both in the Seventh Framework Programme).

### Conflict of interest statement

The authors declare that the research was conducted in the absence of any commercial or financial relationships that could be construed as a potential conflict of interest.

## Footnotes

^1^Note that this is not the same as a vertically flipped ramp. The crucial difference is that the ramp, as the DDM predicts, slowly moves away from baseline from the moment when decision evidence becomes available. For the downramp, on the other hand, there is a sudden break from baseline at the beginning of the decision interval, not consistent with the gradual nature of the evidence accumulation process as predicted by the DDM. At the end of the accumulation process, the ramp sharply drops back to baseline, reflecting the crossing of a decision threshold which ends the accumulation process immediately, while the downramp shows a very gradual return to baseline.
