# Supplementary material for: Is There Neural Evidence for an Evidence Accumulation Process in Memory Decisions?
Source: Front Hum Neurosci. 2016 Mar 8;10:93. doi: 10.3389/fnhum.2016.00093 (PMC4781854; doi:10.3389/fnhum.2016.00093)
Supplement: Supplementary file 1 [file Image1.PDF]

Supplementary Material: Is there neural evidence for an evidence accumulation process in memory decisions?

M. K. van Vugt*, M. A. Beulen, and N. A. Taatgen

*Correspondence:
M. K. van Vugt
m.k.van.vugt@rug.nl

SUPPLEMENTARY TABLES AND FIGURES

Brodmann area	Electrodes	Participants
Amygdala	5	3
Caudate tail	1	1
Hippocampus	26	5
BA 1, 2, 3, 5	17	5
BA 4, 6	28	6
BA 7	12	3
BA 8	13	2
BA 9	27	8
BA 10	37	5
BA 11	16	3
BA 13	1	1
BA 18	12	7
BA 19	32	10
BA 20	153	12
BA 21	117	12
BA 22	53	12
BA 27	3	2
BA 28	24	7
BA 30	4	3
BA 31	1	1
BA 32	1	1
BA 34	6	3
BA 35	26	8
BA 36	27	10
BA 37	26	9
BA 38	59	12
BA 39	19	5
BA 40	53	9
BA 41, 42	16	10
BA 43	7	5
BA 44	11	7
BA 45	18	7
BA 46	16	6
BA 47	14	7

Table S1 Number of electrodes and participants per Brodmann area (BA).

Brain area	Frequency band	Estimate	p-value
BA4,6	delta (2–4Hz)	0.016	0.009119
BA4,6	raw EEG	0.020	0.001239
BA7	theta (4–9Hz)	0.026	0.003278
BA8	delta (2–4Hz)	0.039	5.26e-06
BA18	theta (4–9Hz)	0.051	8.31e-09
BA19	delta (2–4Hz)	0.042	1.16e-12
BA36	theta (4–9Hz)	0.021	0.000891
BA37	alpha (10–14Hz)	0.075	$< 2e-16$
BA39	theta (4–9Hz)	0.026	0.000295
BA40	delta (2–4Hz)	0.013	0.008874
BA40	theta (4–9Hz)	0.013	0.006825

Table S2 Brodmann areas and frequency bands which show significantly higher ($p < 0.05$) correlations with the ramp regressor than average, but where the correlations with downramp and boxcar regressors do not significantly differ from that with the ramp regressor. Estimate shows the deviation of the correlation with the ramp regressor from the grand mean. BA = Brodmann area. BA1,2,3,5 = somatosensory cortex; BA9 = prefrontal cortex; BA19 = occipital cortex; BA37 = occipitotemporal cortex.

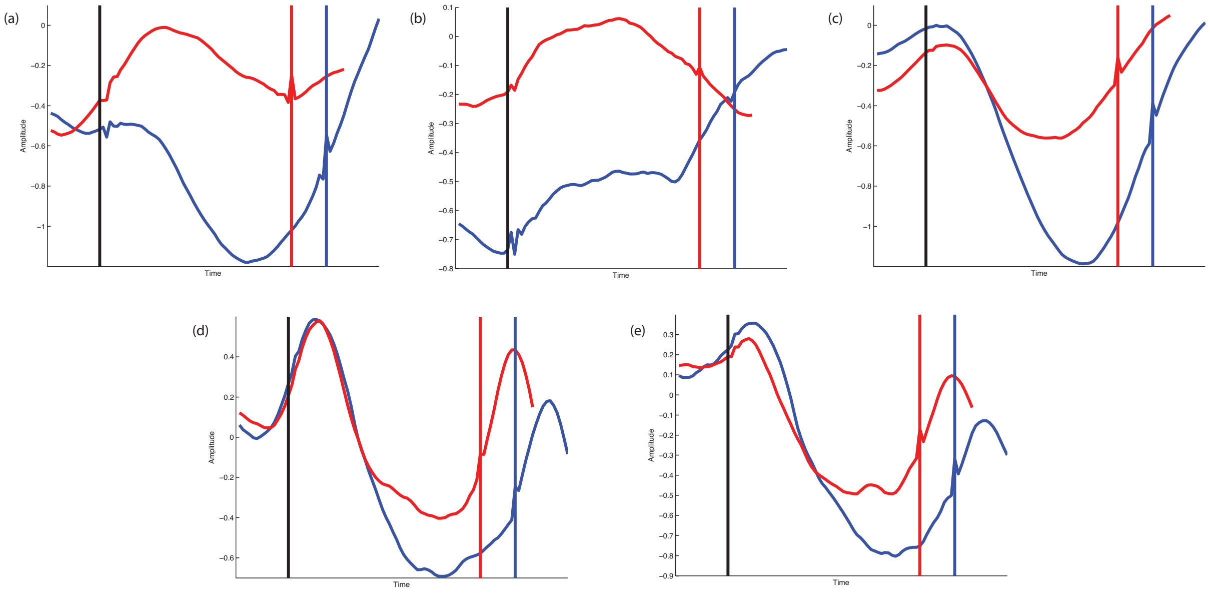

Supplementary Figure 1. Vincentized time course of normalized power for face and letter trials compared.
